# Host-plant-mediated effects of Na*defensin *on herbivore and pathogen resistance in *Nicotiana attenuata*

**DOI:** 10.1186/1471-2229-8-109

**Published:** 2008-10-25

**Authors:** Cbgowda Rayapuram, Ian T Baldwin

**Affiliations:** 1 Department of Molecular Ecology, Max Planck Institute for Chemical Ecology, Hans-Knöll Str. 8, D-07745 Jena, Germany

## Abstract

**Background:**

The adage from Shakespeare, "troubles, not as single spies, but in battalions come," holds true for *Nicotiana attenuata*, which is commonly attacked by both pathogens (*Pseudomonas spp*.) and herbivores (*Manduca sexta*) in its native habitats. Defense responses targeted against the pathogens can directly or indirectly influence the responses against the herbivores. Na*defensin *is an effective induced defense gene against the bacterial pathogen *Pseudomonas syringae *pv *tomato *(PST DC3000), which is also elicited by attack from *M. sexta *larvae, but whether this defense protein influences *M. sexta's *growth and whether *M. sexta*-induced Na*defensin *directly or indirectly influences PST DC3000 resistance are unknown.

**Results:**

*M. sexta *larvae consumed less on WT and on Na*defensin*-silenced *N. attenuata *plants that had previously been infected with PST DC3000 than on uninfected plants. WT plants infected with PST DC3000 showed enhanced resistance to PST DC3000 and decreased leaf consumption by *M. sexta *larvae, but larval mass gain was unaffected. PST DC3000-infected Na*defensin*-silenced plants were less resistant to subsequent PST DC3000 challenge, and on these plants, *M. sexta *larvae consumed less and gained less mass. WT and Na*defensin*-silenced plants previously damaged by *M. sexta *larvae were better able to resist subsequent PST DC3000 challenges than were undamaged plants.

**Conclusion:**

These results demonstrate that Na-defensin directly mediates defense against PST DC3000 and indirectly against *M. sexta *in *N. attenuata*. In plants that were previously infected with PST DC3000, the altered leaf chemistry in PST DC3000-resistant WT plants and PST DC3000-susceptible Na*defensin*-silenced plants differentially reduced *M. sexta's *leaf consumption and mass gain. In plants that were previously damaged by *M. sexta*, the combined effect of the altered host plant chemistry and a broad spectrum of anti-herbivore induced metabolomic responses was more effective than Na*defensin *alone in resisting PST DC3000.

## Background

Plants are attacked in nature by a diverse suite of biotic challenges from pathogens and herbivores which can be devastating. But when plants are attacked by pathogens and herbivores, they mount defense responses which can slow an herbivore's feeding and also the rate of disease spread. For example, in response to herbivore attack, plants produce a broad spectrum of defense compounds that are elicited by a jasmonic acid-dependent signaling pathway. Tomato plants produce potent anti-herbivore defense metabolites such as proteinase inhibitors and polyphenoloxidase when attacked by *Spodoptera exigua *[1]. In response to damage by the solanaceous specialist herbivore *Manduca sexta, Nicotiana attenuata *produces anti-herbivore defense metabolites such as nicotine [2,3], caffeoyl putrescine, rutin, and diterpene glycoside [4], as well as anti-digestive trypsin protease inhibitors (TPIs) [5,6]. On the other hand, plants infected with fungi, bacteria or viruses produce several types of pathogenesis-related proteins (PRs) belonging to at least 17 families [7]. Most PR proteins are known to possess antimicrobial characteristics. For instance, PR-2 (glucanases) of tobacco, barley, alfalfa and soybean have been shown to suppress diseases caused by *Phytophthora megasperma *f. sp. *medicaginins*, *Rhizoctonia solani *and *Alternaria alternata *[7]. PR-3 (chitinases) isolated from bean can suppress *Rhizoctonia solani *in tobacco and canola when overexpressed [8]. *PR-13*/Na-defensins in *N. attenuata *have been shown to suppress *Pseudomonas syrinage *pv*tomato*-DC3000 (PST DC3000) [9].

These examples clearly suggest that depending on the type of the attacker, plants can produce different blends of defense metabolites. But in nature, plants often have to deal with not one but several natural enemies, and these can occur either simultaneously or one after the other, with one enemy facilitating or eliciting resistance to the attack of subsequent attackers. Insects are also well-known vectors for pathogens [10]; moreover, attack by rust fungi can influence subsequent herbivory in the same plant species [11]. The co-evolution of plants and their natural enemies makes it likely that plant responses related to one attacker have far-reaching consequences for subsequent attackers. Plants often produce secondary metabolites with generalized detrimental effects on herbivores as well as pathogens. The phenolic compound rutin is such an example [12]. Constitutive levels of iridoid glycosides among natural variants of *Plantago lanceolata *have been shown to confer resistance to the herbivore *Spodoptera exigua *as well as to the biotropic fungal pathogen *Diaporthe adunca *[11]. Recently it was shown that caterpillar feeding significantly reduced the extent of disease caused by the bacterial pathogens PST DC3000 and *Xanthomonas campestris *pv*armoraciae *in Arabidopsis [13].

Although many studies have shown that pathogen- and herbivore-elicited plant responses can have detrimental effects on subsequent attacker(s), few have established causal associations with the induction of a specific metabolite(s) by the first attacker with a detrimental effect (direct or indirect) on subsequent attacker(s). Some studies have causally linked the signaling pathways that mediate effects against herbivores and pathogens [13], others have found associations among the amounts of constitutively expressed metabolites (iridoid glycosides) known to affect both herbivores and pathogens; but all these studies have been conducted with genotypes selected over generations for high- and low-leaf iridoid glycoside concentrations [11] and are not iso-genic. Therefore, they may differ in resistance traits other than the measured metabolites. As with constitutively produced metabolites, little is known about the cross effects of inducible defense metabolites.

Here we 1) identify a defense metabolite (Na-*defensin*) in WT *N. attenuata *that is elicited in response to attack from both pathogens and herbivores and 2) examine the consequences of this metabolite for both pathogen and herbivore resistance by comparing the resistance of WT and iso-genic plants transformed to silence the expression of the metabolite. *N. attenuata*, a solanaceous annual originating from the Great Basin Desert or North America, dramatically increases Na*defensin *(also known as *PR-13*) levels when attacked by herbivores such as *Manduca sexta *larvae [14,15], *Tupiocorus notatus, Myzus nicotianae, Spodoptera littoralis *and *Trichoplusia ni *[16] as well as the pathogen PST DC3000 [9]. Previous work from our lab has shown that WT *N. attenuata *plants silenced for Na*defensin *made WT plants increasingly susceptible to PST DC3000 [9]. Our main objectives were to study 1) the role of defensin in *N. attenuata*'s induced resistance to *M. sexta *and 2) the influence of defensin on *M. sexta *growth when elicited by PST DC3000 and vice versa. We began by observing the patterns of Na*defensin *expression in *N. attenuata *in response to *M. sexta *and PST DC3000 and then studied the effect of Na*defensin *on *M. sexta's *resistance to plants that had been previously infected with PST DC3000 as well as the effects of *Nadefensin *on PST DC3000 resistance when plants had been previously damaged by *M. sexta*.

## Results

### Gene expression analysis of Na*defensin *in WT and transgenic *N. attenuata *plants silenced for Na*defensin*

In *N. attenuata*, Na*defensin *(NCBI accession AY456268) is up-regulated in WT *N. attenuata *plants after attack from *M. sexta *[14,15], *Tupiocorus notatus, Myzus nicotianae, Spodoptera littoralis *and *Trichoplusia ni *[16] larvae. Bacteria (*Pseudomonas syringae*) are also known to induce *defensin *in different plants [17,18]. Recently, we reported that Na*defensin *was up-regulated 12 h after WT *N. attenuata *plants were infected with PST DC3000 [9]; moreover, silencing Na*defensin *by RNAi by stable transformation (ir*defensin *lines 76 and 96) increased the plant's susceptibility to PST DC3000 [9].

**Figure 1:**
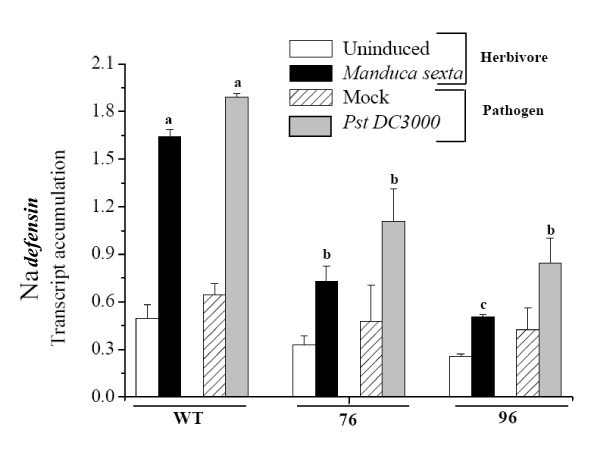
***M. sexta *damage and Pst DC3000 inoculation increase Na*defensin *transcripts; responses are highly attenuated in ir*defensin *(76 and 96) lines**. Quantitative real-time PCR (qRT-PCR) was used to analyze Na*defensin *transcript accumulation in WT *N. attenuata *plants and ir*defensin *(76 and 96) lines in response to continuous *M. sexta *feeding by first-instar larvae for 4 days or inoculation with *Pseudomonas syringae *pv *tomato *DC3000 (Pst DC3000) (1 × 10^5 ^cells/ml). Values are mean (± SE) Na*defensin *transcripts from 3 replicate plants per treatment normalized to the transcript abundance of *actin*, which is unregulated under these conditions. Different letters indicate significant differences between genotypes damaged by *M. sexta *and infected by Pst DC3000.

While attack from both *M. sexta *larvae and PST DC3000 is known to elicit Na*defensin *transcripts and protein in *N. attenuata*, the relative responses to *M. sexta *larvae and PST DC3000 challenges were not known. We re-examined the levels of Na*defensin *transcripts accumulation in PST DC3000 and *M. sexta*-attacked plants at a single time point (4 days after pathogen and herbivore damage). The quantitative real-time PCR (qRT-PCR) analysis (Fig. 1) revealed that Na*defensin *transcript accumulation differed significantly across treatments and genotypes (Fig. 1; ANOVA *F*_11,17 _= 16.00, *P *< 0.001): Na*defensin *levels in WT plants infected with PST DC3000 and in those attacked by *M. sexta *did not differ significantly (Fig. 1; *p *= 0.183). Consistent with our earlier observation [9], WT plants either damaged by *M. sexta *or infected by PST DC3000 had significantly more (at least 60%) Na*defensin *transcripts compared to similarly treated ir*defensin *plants (76 and 96). The similar levels of Na*defensin *after *M. sexta *damage or PST DC3000 infection suggest that Na*defensin *is likely elicited by jasmonates which are produced in response to pathogen infection as well as insect attack [19].

### Effects of PST DC3000 infection and Na*defensin *silencing on herbivore performance

Since Na*defensin *is expressed in response to attack from both herbivores and pathogens, we asked if silencing Na*defensin *expression influenced *M. sexta *growth in uninduced plants as well as in plants previously inoculated with PST DC3000. We carried out assays on WT and Na*defensin*-silenced plants (lines 76 and 96) which were either uninduced or had been infected (4 days earlier) with PST DC3000. We measured two parameters that reflect the overall performance of *M. sexta *larvae: percentage of leaf area damage and larval mass gain.

### Percentage of leaf area damage

**Figure 2:**
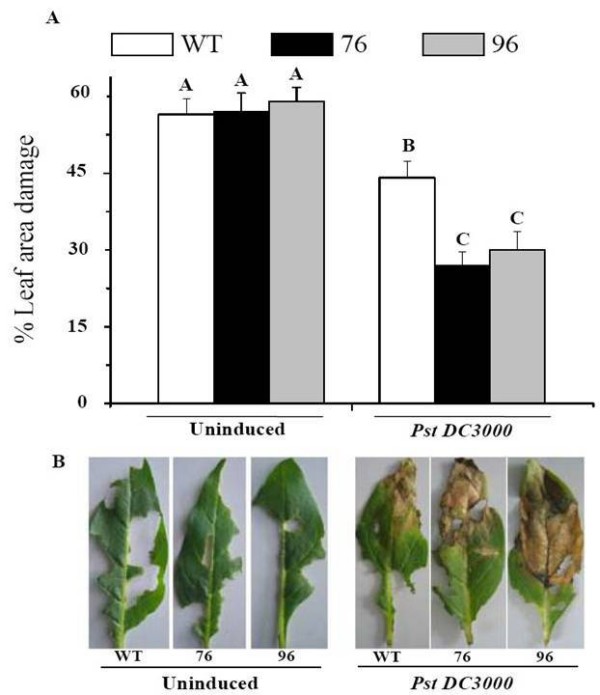
**Pst DC3000 inoculation and Na*defensin *silencing decreases leaf area damage by *M. sexta *larvae in *N. attenuata***. **A) **Mean (± SE) percentage of leaf area damage by *M. sexta *larvae on WT plants and ir*defensin *lines 76 and 96. A neonate larva was placed in a clip cage and allowed to feed for 12 days before the percentage of leaf area damage was estimated. **B) **Photographs taken after 12 days of *M. sexta *feeding on WT and ir*defensin *lines 76 and 96 that were either uninduced (left) or inoculated with Pst DC3000 (right). Different letters indicate significant differences between treatments and genotypes (N = 18).

After 12 days of attack from a single *M. sexta*, leaves were evaluated for the percentage of leaf area damaged. *M. sexta *larvae removed significantly more leaf area from uninduced WT and ir*defensin *(76 and 96) plants (at least 30%) than from PST DC3000-infected plants (Fig. 2A and 2B; ANOVA, *F*_5,88 _= 19.67, *P *< 0.001). Within the uninduced treatment, no significant differences in the percentage of leaf area damage between WT and ir*defensin *line 76 (*p *= 0.905) plants or between WT and ir*defensin *line 96 (*p *= 0.517) plants were observed (Fig. 2A). On the other hand, prior infection with PST DC3000 resulted in greater leaf area losses (at least 35%) in WT plants compared to plants from both ir*defensin *lines (Fig. 2A and 2B; line 76 *p *< 0.001; line 96 *p *= 0.002).

### Larval mass gain

**Figure 3:**
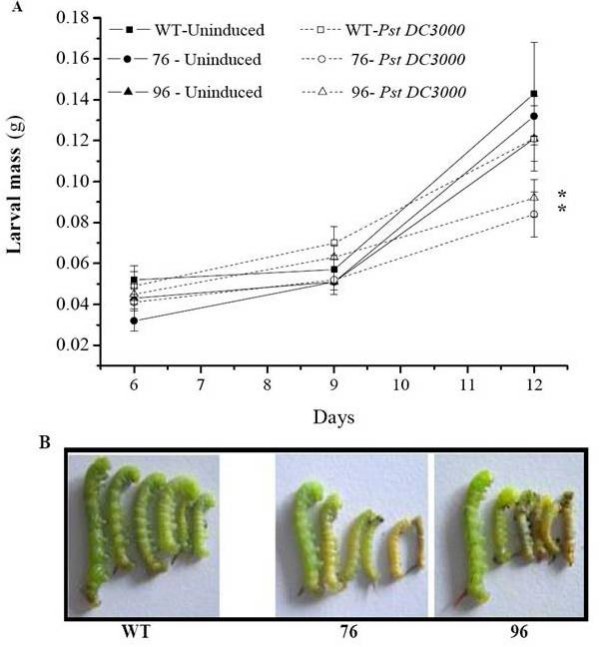
**Pst DC3000 inoculation and Na*defensin *silencing decrease *M. sexta *larval mass gain in *N. attenuata***. **A)**. Mean (± SE) *M. sexta *larval mass gain on WT plants and ir*defensin *lines 76 and 96. A neonate larva was placed in a clip cage and allowed to feed continuously for 12 days. Larval mass was recorded on days 6, 9 and 12. **B) **Photographs taken after 12 days of *M. sexta *feeding on WT and ir*defensin *lines 76 and 96 that were induced with Pst DC3000. Asterisk indicates significant differences (p = 0.05) between WT and ir*defensin *lines (76 and 96) after Pst DC3000 infection (N = 30).

We also measured the mass of the larvae that fed on uninduced and on PST DC3000-infected WT and ir*defensin *(76 and 96) plants. ANOVA revealed significant differences among the treatments and the genotypes (Fig. 3A and 3B; ANOVA, *F*_17,426 _= 14.14, *P *< 0.001), but the larval mass differences differed from those of the pattern leaf area damaged. No significant differences in larval mass between the *M. sexta *larvae that fed on the uninduced WT and those that fed on WT plants which were PST DC3000 infected was observed (*p *= 0.264). Within the uninduced treatment, no statistical differences in the mass of larvae that fed on WT and ir*defensin *line 76 (*p *= 0.427) plants or WT and ir*defensin *line 96 (*p *= 0.117) plants were observed (Fig. 3A). On the other hand, larvae that fed on WT plants infected with PST DC3000 gained significantly more (at least 70%) mass than did larvae that fed on infected plants from ir*defensin *line 76 (p = 0.012) and ir*defensin *line 96 (p = 0.045). The larvae that fed on PST DC3000-infected ir*defensin *(76 and 96) plants were smaller than the larvae that fed on PST DC3000-infected WT plants (Fig. 3B). The large variation in the larval mass across the experiment could be attributed to the differences in larvae's development which in turn may be attributed to high spatial heterogeneity in food quality for the larvae consuming infected leaves.

### Detecting PST DC3000 from infected plants in herbivores' guts

**Figure 4:**
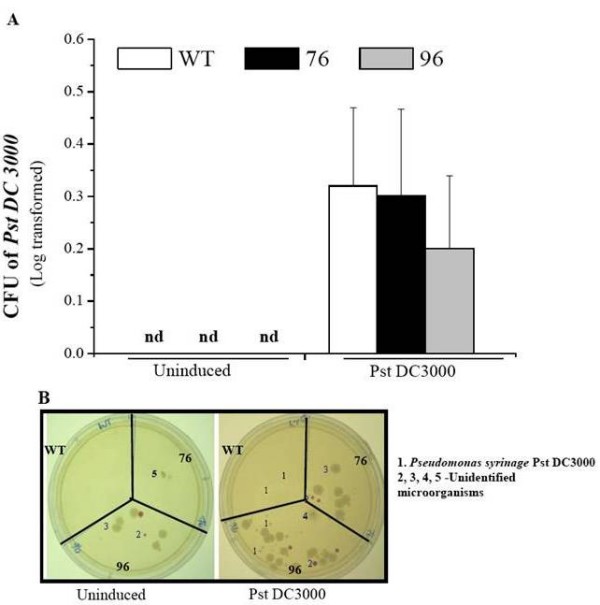
**The number of Pst DC3000 colonies quantified in the guts of *M. sexta *larvae that fed on the Pst DC3000-inoculated WT and ir*defensin *(76 and 96) plants do not differ**. **A) **Mean (± SE) colony-forming units (CFUs) of Pst DC3000 in the guts of larvae that fed on either the Pst DC3000-inoculated WT/ir*defensin *(76 and 96) or uninduced WT/ir*defensin *(76 and 96) plants. The larval guts from 5 replicate larvae that fed on either Pst DC3000-inoculated WT and ir*defensin *(76 and 96) for 12 days were surgically removed and ground in 1 ml sterile water. 40 μl of supernatant was spread on plates containing LB agar plate containing rifamycin and tetracycline to select for the growth of Pst DC3000. Colonies were counted after 48 h of incubation at 28°C. **B) **Photographs of LB plates + antibiotics (rifamycin and tetracycline) showing Pst DC3000, in addition to four unidentified/unknown microorganisms that could also grow on LB plants supplemented with antibiotics (N = 5).

In our earlier work, we reported that ir*defensin *(76 and 96) plants were more susceptible to PST DC3000 than were WT *N. attenuata *plants, and as a result ir*defensin *(76 and 96) plants contained more PST DC3000 colony forming units (CFUs) than did the WT plants [9]. In this study we observed that *M. sexta *larvae that fed on ir*defensin *(76 and 96) plants were smaller and seemed to be infected with pathogens (Fig. 3B). Therefore, we hypothesized that herbivores feeding on PST DC3000-infected ir*defensin *(76 and 96) plants might have ingested more PST DC3000 than did the larvae feeding on the PST DC3000-infected WT plants, and that the number of ingested PST DC3000 might negatively correlate with larval growth. We counted the CFUs of plant-derived PST DC3000 in guts (including the foregut, midgut and hindgut) of larvae that fed either on PST DC3000-infected WT plants or PST DC3000-infected ir*defensin *plants (76 and 96) (Fig. 4A and 4B). As expected, we found PST DC3000 colonies in larvae that fed on PST DC3000-infected WT and ir*defensin *(76 and 96) but none in larvae that fed on uninfected WT and ir*defensin *(76 and 96) plants. However, the number of PST DC3000 colonies in the guts of larvae that fed on PST DC3000-infected WT or ir*defensin *(76 and 96) plants did not differ significantly (Fig. 4A; ANOVA, *F*_5,24 _= 2.07, *P *= 0.104). Moreover, the overall number of CFUs was very low relative to the number of CFUs found in leaves, which suggests that plants infected with PST DC3000 do not detrimentally affect larvae by directly transmitting pathogens to the herbivores. In addition to PST DC3000, we also detected a few unknown microorganisms with resistance to tetracycline and rifamycin (the selection markers for PST DC3000). Interestingly these unknown microorganisms were found most often in guts extracted from larvae that fed on ir*defensin *(76 and 96) plants. ir*defensin *(76 and 96) plants also show an increased susceptibility to the many opportunistic microorganisms which may be detrimental to larvae as well (Fig. 4B).

### Effects of herbivory and Na*defensin *silencing on PST DC3000 infection

**Figure 5:**
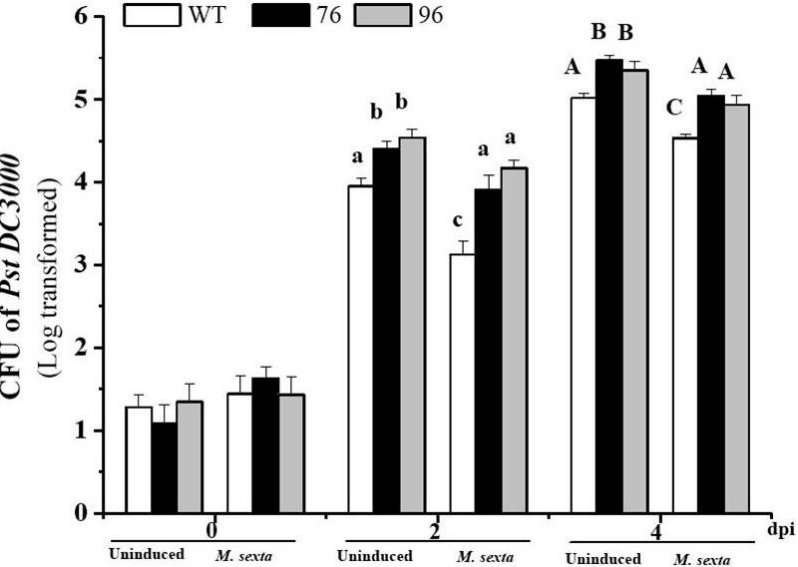
***M. sexta *feeding significantly reduces Pst DC 3000 disease spread**. Values are mean (± SE) colony-forming units (CFUs) of Pst DC3000 after inoculation of the leaves of WT/ir*defensin *(76 and 96) plants that were either uninduced or previously attacked by *M. sexta *larvae 4 days earlier. To record the CFUs, surface-sterilized leaf discs (1 cm^2^) were ground in 1 ml sterile water and 40 μl of supernatant was spread on plates containing LB agar + antibiotics (rifamycin and tetracycline). Colonies were counted after 48 h of incubation at 28°C. Different letters in lower and upper cases indicate significant differences among Pst DC3000-inoculated WT plants and the transgenic plants on days 2 and day 4, respectively (N = 5).

Silencing Na*defensin *expression in *N. attenuata *does not influence the plant's resistance to *M. sexta *attack but lowers resistance to PST DC3000 [9], which suggests that Na*defensin *functions as an antibacterial defense protein in *N. attenuata*. We therefore explored whether Na*defensin *still functions as an antibacterial protein in leaves that are damaged by herbivores. We compared the level of disease progression of PST DC3000 in leaves that were either undamaged or previously damaged (4 days) by *M. sexta*. Two and four days after PST DC3000 infection, leaves were evaluated for CFUs. In general, we found that inoculating leaves of undamaged plants with PST DC3000 or infecting leaves of *M. sexta*-damaged (4 days of feeding) plants with PST DC3000 resulted in statistically significant differences in PST DC3000 growth responses in *N. attenuata *(Fig. 5; ANOVA, *F*_17,72 _= 128.75, *P *< 0.001). Investigating the genotypic and treatment effects, we found the following patterns on day 4: 1) PST DC3000 CFUs were higher in both uninduced ir*defensin *line 76 (9%; *p *= 0.031) and line 96 (6.6%; *p *= 0.047) than in uninduced WT plants; 2) similarly, PST DC3000 CFUs were higher in *M. sexta*-damaged plants from both ir*defensin *lines 76 (11.3%; *p *= 0.014) and line 96 (8.9%; *p *= 0.048) than in WT plants; 3) within the WT plants, control plants (undamaged) had higher titers of PST DC3000 CFUs (10.6%; *p *= 0.017) than did *M. sexta*-damaged plants; 4) within the ir*defensin *lines (76 and 96) the same effects of *Manduca *damage were observed: control plants (undamaged) had a higher titers of PST DC3000 CFUs, 8.53% (*p *= 0.037) and 8.51% (*p *= 0.0183), respectively, than did the *M. sexta*-damaged lines; 5) control WT plants (T4-undamaged) and *M. sexta*-damaged ir*defensin *lines 76 and 96 did not differ in PST DC3000 CFUs (*p *= 0.934 and *p *= 0.676, respectively). In summary, these results suggest that Na-defensin's anti-bacterial defense property is retained in leaves regardless of whether a leaf is elicited by pathogen or herbivore. In addition, *M. sexta *damage which results in the elicitation of a large set of anti-herbivory defense metabolites more effectively restricted PST DC3000 growth than did elicitation by Na-defensin alone.

## Discussion

Two observations motivated us to study the direct and indirect roles of Na*defensin *in resistance to pathogens and herbivores in *N. attenuata*: 1) Na*defensin *levels are increased in *N. attenuata *in response to attack from these two natural enemies and 2) Na*defensin *is effective in resisting PST DC3000 in *N. attenuata *but not herbivores [9]. Therefore we asked: Does Na*defensin *(an anti-bacterial defense gene) have a function during herbivore attack? This question led us to ask if Na*defensin *indirectly affects interactions between *M. sexta *and PST DC3000.

The results demonstrate that PST DC3000-infection significantly reduces *M. sexta's *leaf consumption and growth; its effects are most dramatically seen in the Na*defensin*-silenced plants. Since Na*defensin*-silenced plants are more susceptible than WT plants to PST DC3000 [9], we inferred that either the presence of PST DC3000 in the larval diet or the altered leaf chemistry resulting from PST DC3000 infection in Na*defensin*-silenced plants was responsible for the poor larval performance. Larvae that fed on PST DC3000-infected WT plants, which are resistant to PST DC3000, consumed significantly more than those that fed on PST DC3000-infected Na*defensin*-silenced plants, which are highly susceptible to PST DC3000 (Fig. 2A and 2B); however, we found no difference in the titers of PST DC3000 in the guts of the larvae (Fig. 4A and 4B) that had consumed either WT or Na*defensin*-silenced plants. We propose that changes in plant chemistry associated with differences in PST DC3000 resistance negatively affect leaf consumption, but not the pathogen *per se*. This scenario is consistent with earlier studies in which tomato leaflets that had an increase in polyphenol oxidase after being infected by PST DC3000 decreased the suitability of non-inoculated leaflets of the same leaf for *Helicoverpa zea *[1]. Interestingly, our results show that despite differences in leaf consumption between *M. sexta *that fed on either uninduced WT or Na*defensin*-silenced plants and those that fed on PST DC3000-infected WT plants, larvae nevertheless gained the same amount of body mass (Fig. 3A). Since body mass is maintained at lower levels of consumption, PST DC3000 infection of WT plants appears to increase the efficiency by which ingested food creates body mass for *M. sexta *larvae.

We noticed that larvae avoided consuming the portions of the leaf exhibiting disease symptoms (Fig. 2B) and found leaf consumption to be inversely related to the size of the disease lesions. Why do larvae feeding on PST DC3000-infected WT plants gain more mass even when they consume less? Plant responses to pathogen infection such as increased salicylic acid (SA), which is produced in response to PST DC3000 infection, could be involved [9]. Several studies have shown that growth in herbivores is enhanced in plants that have elevated SA levels because SA can antagonize the oxylipin signaling which mediates herbivore resistance [20-22]. However, WT and Na*defensin*-silenced plants infected by PST DC3000 have the same level of SA [9], so other unmeasured changes in leaf chemistry are likely involved.

Na*defensin *can function as an anti-bacterial protein and is capable of inhibiting PST DC3000 growth. Since Na*defensin *is also induced after *M. sexta *damage [14,15], we hypothesized that after herbivore damage Na*defensin*-silenced plants should also be susceptible to PST DC3000. Indeed, Na*defensin*-silenced plants that were uninduced or previously damaged by *M. sexta *were more susceptible to PST DC3000 than the WT plants that were uninduced or previously damaged by *M. sexta*, respectively. Clearly, Na-defensin functions as an anti-bacterial defense protein regardless of whether it is elicited by either pathogens or herbivores. Moreover, it appears that herbivore-mediated cellular changes do not affect Na*defensin's *known anti-bacterial function. This is not surprising given the structure of the protein. Na*defensin *belongs to the γ-Na-defensin class, which is 40–45 amino acids long, with 8 conserved cysteine residues that form 4 disulphide bridges; these disulphide bridges are thought to contribute to the stability of the protein. Moreover, Na-defensin is a cationic-basic protein and thus can be attracted to bacterial or fungal cell membranes, where it interacts directly [23]. We do not know exactly what feature of plant responses (other than jasmonates) towards pathogens or herbivores causes the induction of Na*defensin*, but plants may elicit Na*defensin *to prime their defense responses to possible bacterial damage. The wounds that herbivore feeding causes are likely entry points for many pathogens. This scenario is consistent with the lower PST DC3000 growth in WT or Na*defensin*-silenced plants that were previously fed on by *M. sexta *compared to WT or Na*defensin*-silenced plants that were previously undamaged. *M. sexta*-damaged plants had reduced PST DC3000 CFUs, which is consistent with other studies reporting that herbivore-damaged plants may be less suitable for pathogens [24,25]. Herbivore damage is associated with the production of several defense metabolites that can have detrimental effects on herbivores as well as pathogens. For instance, *M. sexta *damage increases nicotine production in *N. sylvestris*[26] and *N. attenuata *[3], and nicotine was found to inhibit the growth of five species of *Pseudomonas *bacterial pathogens [12]. Similarly, many phenolic compounds such as rutin and chlorogenic acid are also produced in *N. attenuata *[4]. Rutin, for instance, is a broad spectrum defense metabolite [12]. Accordingly, after herbivory plants likely elicit herbivore-specific metabolites as well as anti-bacterial defense proteins such as defensin. The latter may be elicited in anticipation that the wounds created during herbivory might provide an opening for pathogenic bacteria.

## Conclusion

We studied the three-way interaction between a plant (*N. attenuata*), an herbivore (*M. sexta*) and pathogenic bacteria (PST DC3000) with reference to a known anti-bacterial defense protein, Na-defensin. Na-defensin was found to be effective in containing PST DC3000 growth but ineffective in preventing *M. sexta *from feeding and gaining body mass. When the indirect effects of Na*defensin *on herbivore performance were analyzed in PST DC3000-infected WT leaves, we found that the increased expression of Na*defensin *increased resistance to PST DC3000 and also reduced *M. sexta's *leaf consumption but not its larval mass gain. Reduced Na*defensin *expression in PST DC3000-infected Na*defensin*-silenced plants decreased resistance to PST DC3000 but hindered *M. sexta's *leaf consumption and its mass gain. When we analyzed the Na*defensin*-mediated responses in *M. sexta-*damaged leaves to PST DC3000 resistance, we found that damage by *M. sexta *increased the resistance of both WT and Na*defensin*-silenced plants to PST DC3000. This enhanced resistance in *M. sexta*-damaged plants may result from the elicitation of a large set of anti-herbivory defense metabolites that can affect both herbivores as well as pathogens and to some specific anti-bacterial defense proteins such as defensin.

## Methods

### Plant material, *Pseudomonas syringae *growth, plant treatments

Wild-type (WT) *N. attenuata *plants (seeds collected from a native population from the DI Ranch, Santa Clara, UT, USA) that had been inbred for 14 generations were used in this study. Transgenic plants, ir*defensin *lines (76 and 96), were produced in the same WT genetic background. Germination was carried out according to the procedures described in [27]. Plants were grown in chambers (16/8 hr photoperiod at 25/21°C, and 45–55% relative humidity) and experiments were carried out with rosette-stage plants 14 days after they were transferred to 1 l pots.

The virulent strain of *Pseudomonas syringae *pv *tomato*-DC3000 (PST DC3000) strain was grown and maintained on a LB agar plates at 28°C. The PST DC3000 growth and inoculation procedure was carried out as described in [28]. In brief, 1 × 10^5 ^cells/ml were resuspended in 0.1% Silwett L-77 solution and intact leaves were dipped for 1 minute. As a mock inoculation, leaves were dipped in 0.1% Silwett L-77 solution.

### Isolating Na*defensin*, and generating and characterizing Na*defensin*-silenced plants

Na*defensin *was identified as a differentially regulated gene in WT *N. attenuata *plants that had been damaged by the specialist herbivore *M. sexta*. The sequence has been submitted in the NCBI database (accession number AY456268) [14]. Using a forward primer (FLTIO-FP: 5'ATGGCTCGATCCTTGTGCTTCATG 3' and a reverse primer FLTIO-RP: 5'TTAGTTATCCATCATCTCTTC 3'), an Na*defensin *sequence was PCR amplified from the cDNA obtained from PST DC3000-inoculated WT leaves and this sequence was used to generate transgenic plants ir*defensin *lines (76 and 96) as described in [9]. In brief, a 225 bp fragment from Na*defensin *ORF was inserted into a pRESC5 transformation vector to create an inverted-repeat (ir) construct. These constructs were transformed into *N. attenuata *WT plants using an *Agrobacterium*-mediated transformation procedure described in [27]. The gene for hygromycin resistance (*hptII*) allowed transformed plants to be identified easily by selecting hygromycin-resistant individuals [27].

### Nucleic acid analysis

#### Transcript analysis

To analyze Na*defensin *transcripts, we extracted total RNA with TRIzol reagent (Invitrogen, ) following the TIGR protocol ). cDNA was synthesized from 1 μg RNA using the SuperScript™ II RT enzyme (Invitrogen) as described in [29]. The transcripts were analyzed by quantitative real-time PCR (ABI PRISM™7000, Applied Biosystems,  which was conducted using the qPCR™ core reagent kit (Eurogentec, ). To analyze Na*defensin*, a specific TaqMan primer pair (forward primer: 5'-AACTATGGCTCG CTCCTTGTGC-3', the reverse primer: 5'-CTCATAGGCAACAAAAAGCAT-3') and a double fluorescent dye-labeled probe (5'-TTCATGGCATTTGCAGTCTTGGCAA-3') were used. The relative gene expression was calculated using a 10-fold dilution series of cDNAs which had been transcribed from induced RNA samples from the same experiment.

### Analysis of herbivory

#### Leaf area damage and larval mass

We placed 5-day-old larvae that were previously reared on WT *N. attenuata *leaves individually on the fully developed leaves of rosette-stage WT and ir*defensin *lines (76 and 96) that were either pre-infected with PST DC3000 or left uninfected (N = 30). Each larva was enclosed in a well-aerated 5 cm diameter clip cage. The larvae were weighed 6, 9 and 12 days after feeding. Leaf area damage was estimated at the end of 12 days and based on the extent of leaf damage, with values ranging from 1 to 5 [1(0–15%), 2(16–30%), 3(31–45%), 4(46–60%), 5(61–75%) and 7(> 76%)] was assigned.

### Analysis of pathogen growth

To quantify the disease spread in WT and ir*defensin *lines (76 and 96) plants, we quantified the colony forming units (CFUs) in PST DC3000 inoculated leaves. In brief, surface-sterilized leaf discs (1 cm^2^) were ground in 1 ml sterile water and 40 μl of supernatant was spread on plates containing LB agar containing rifamycin and tetracycline. Colonies were counted after 48 h of incubation at 28°C.

### Statistical analysis

Data were analyzed with StatView (Abacus Concepts, Inc., ).

## Authors' contributions

CR carried out the molecular studies, herbivore and pathogen bio-assays and statistical analysis. ITB designed and coordinated the experiments and also helped to draft the manuscript. CR and ITB read and approved the final manuscript
